# Inhibiting CK2 among Promising Therapeutic Strategies for Gliomas and Several Other Neoplasms

**DOI:** 10.3390/pharmaceutics14020331

**Published:** 2022-01-30

**Authors:** Emanuela B. Pucko, Robert P. Ostrowski

**Affiliations:** Department of Experimental and Clinical Neuropathology, Mossakowski Medical Research Institute, Polish Academy of Sciences, 5 Pawińskiego St., 02-106 Warsaw, Poland; epucko@imdik.pan.pl

**Keywords:** gliomas, kinase CK2, benzimidazoles, isothioureas, hyperbaric oxygen

## Abstract

In gliomas, casein kinase 2 (CK2) plays a dominant role in cell survival and tumour invasiveness and is upregulated in many brain tumours. Among CK2 inhibitors, benzimidazole and isothiourea derivatives hold a dominant position. While targeting glioma tumour cells, they show limited toxicity towards normal cells. Research in recent years has shown that these compounds can be suitable as components of combined therapies with hyperbaric oxygenation. Such a combination increases the susceptibility of glioma tumour cells to cell death via apoptosis. Moreover, researchers planning on using any other antiglioma investigational pharmaceutics may want to consider using these agents in combination with CK2 inhibitors. However, different compounds are not equally effective when in such combination. More research is needed to elucidate the mechanism of treatment and optimize the treatment regimen. In addition, the role of CK2 in gliomagenesis and maintenance seems to have been challenged recently, as some compounds structurally similar to CK2 inhibitors do not inhibit CK2 while still being effective at reducing glioma viability and invasion. Furthermore, some newly developed inhibitors specific for CK2 do not appear to have strong anticancer properties. Further experimental and clinical studies of these inhibitors and combined therapies are warranted.

## 1. Introduction

For many decades, much attention has been given to the mechanisms of protein kinases in cellular processes. Protein phosphorylation is a recurring topic in medical science as the mechanism that regulates various cellular processes. Protein modification is catalysed by protein kinases that use ATP as the source of phosphate [1,2]. Protein kinases have become the aim of targeted therapy because they play a key role in proliferation, cell cycle, and cell death. Unfortunately, mutations and amplification of genes encoding kinases disrupt many signalling pathways, resulting in numerous diseases, including malignant neoplasms. Therefore, protein kinases have become a priority in research work of scientific centres and pharmaceutical companies around the world [3]. Chemical compounds that can regulate or inhibit kinases, and consequently prevent diseases, are being sought. Ideally, these would be specific inhibitors assigned to a particular kinase, in which case the inhibitor would serve as the target of personalized treatment. One of the milestones in cancer treatment turned out to be the chemical inhibition of protein kinases and the use of kinase inhibitors in the fight against cancer. Particularly successful was imatinib (Glivec; Gleevec in the USA), one of the first small molecule inhibitors of oncogenic tyrosine kinase, registered by the FDA in 2001. The discovery of imatinib changed the approach to treatment and at the same time started the expansion of kinase inhibitors as a new class of drugs [4]. 

## 2. Neoplasms and the Significant Role of CK2 in Tumour Biology

### 2.1. Glioblastoma 

Numerous scientific studies have suggested that it is worth identifying new classes of protein kinase inhibitors with different mechanisms of action and different target points. One such kinase is the protein kinase CK2, which has been found to play a role in the viability and invasiveness of several neoplasms. Among those, glial neoplasms constitute the largest group of tumours in the central nervous system, and glioblastoma (per WHO G4, the fifth edition of the WHO Classification of Tumours of the Central Nervous System) is the representative of this group, carrying the highest degree of malignancy [5]. Glioblastomas comprise 16% of all primary brain tumours and is most often diagnosed in middle-aged patients [6]. American statistics have indicated that 3.19 people per 100,000 suffer from malignant gliomas every year [7]. The classification introduced by the World Health Organization (WHO) distinguishes four degrees of histological malignancy of astroglial tumours. In the first grade are benign tumours with a positive prognosis for patients. In contrast, histological high-grade tumours are characterized by rapid cell proliferation, infiltrating growth, and, unfortunately, poor prognosis [8,9].

In case of the glioblastoma, a surgical resection, radiotherapy and chemotherapy with temozolomide, lomustine, and numerous experimental methods are used [10]. For this reason, the priority is to identify effective therapies that are capable of eliciting sustained responses in patients. Unfortunately, most patients experience a relapse and die within a few months. The reasons for the ineffectiveness in the treatment of gliomas may rest with the blood–brain barrier (BBB), the tumour microenvironment, mutations in genes encoding kinases, overexpression of growth factors, and a subpopulation of glioblastoma stem cells (GSCs) [11,12,13,14,15]. Experimental studies have suggested that it is GSCs that may contribute to, and at the same time justify, tumour regrowth and metastasis [16]. In the diagnosis of tumours of the central nervous system (CNS), the WHO has introduced a classification in which genetic abnormalities are of utmost importance, although remaining wedded to clinical and pathological features of gliomas. Mutations within the genes encoding IDH1 or IDH2 dehydrogenase isocitrate and several other genes, including *EGFR* (epidermal growth factor receptor), *pTERT* (telomerase reverse transcriptase promoter)*, CDKN2A/B* (cyclin-dependent kinase inhibitor 2A/B), and *BRAFV600* (mutation of the B-Raf gene in which valine is substituted by glutamic acid at amino acid 600) are fundamental in this classification. In general, IDH-mutated gliomas belong to grades 2–4, while IDH wild type is mainly grade 4. The WHO classification of 2021 removed the term IDH-mutant glioblastoma, ascribing this mutation to astrocytic tumours grades 2–4 (G2–4) designated by Arabic numerals [5]. CK2 levels have been found elevated in glioblastoma biopsy samples. Moreover, CK2 regulates glioma cell viability and confers resistance to TNFα-induced apoptosis [17].

### 2.2. Medulloblastoma 

Medulloblastoma (MB) is a malignant brain tumour in children and accounts for approximately 20% of all paediatric central nervous system (CNS). Treatment of medulloblastoma has led to a 70–90% five-year overall survival rate, but the prognosis for patients with tumour dissemination and recurrent neoplasm MB remains poor, and the majority of survivors exhibit long-term neurocognitive and neuroendocrine complications as a result of the cytotoxic drugs and radiation. CK2 contributes to MB tumorigenesis, and exogenous expression of CK2 enhances MB cell growth and tumour growth in vivo [18].

### 2.3. Acute Lymphoblastic Leukaemia 

Acute lymphoblastic leukaemia (ALL) is derived from B- and T-lymphoid progenitors. Identification is based on morphologic, immunophenotypic, and genetic characteristics. Chemotherapy regimens have accomplished overall cure rates of 40 to 50% in adults and 85 to 90% in children [19]. Unfortunately, ALL is the most common child malignancy, and despite an eighty percent cure rate, relapsed disease remains the leading cause of mortality. Associated mutations in genes and transcriptional dysregulations may lead to chemotherapy resistance, higher-risk ALL, and poorer prognosis [20]. CK2 overexpression has been demonstrated in acute lymphoblastic leukaemia. In this context, CK2 is a prosurvival kinase contributing to resistance to chemotherapy, hence being a potential therapeutic target [21].

### 2.4. Chronic Lymphocytic Leukaemia 

Chronic lymphocytic leukaemia (CLL) is a disease of aging adults. The disease results from the overgrowth of a single CD5+ B lymphocyte coexpressing low levels of surface membrane immunoglobulin (smIg); a single IG light (L) chain type; and CD79b, CD20, and CD23. The clinical consequences of this clonal overgrowth are highly variable: some patients die within 2 years of diagnosis, whereas others survive several years. This variability is due to factors intrinsic to the leukemic B cell, genetic and epigenetic changes in genes, and factors extrinsic to the leukemic cell (e.g., inputs delivered by various signalling pathways in the tissue microenvironment) [22]. CK2 has been found overexpressed and hyperactivated in primary CLL cells from untreated patients and has been postulated to play an important role in the biology of CLL [23]. 

### 2.5. Acute Myeloid Leukaemia 

Acute myeloid leukaemia (AML) is the most common acute leukaemia in adults, and prognosis varies widely. AML is a highly heterogeneous disease caused by chromosomal translocations and mutations in the genes involved in hematopoietic proliferation and differentiation, which result in the accumulation of poorly differentiated myeloid cells. The backbone of therapy remains a combination of cytarabine- and anthracycline-based regimens with allogeneic stem cell transplantation for eligible candidates, but elderly patients are often unable to tolerate such regimens and carry a particularly poor prognosis [24]. Protein kinase CK2 has been found to play pivotal roles in AML biology, and targeting CK2 has emerged as viable therapeutic option [25].

### 2.6. Acute Promyelocytic Leukaemia 

Acute promyelocytic leukaemia (APL) accounts for 10–15% of all acute myeloid leukaemias and is characterized by a block in differentiation during which leukemic cells are halted at a distinct stage in cellular maturation, specifically the promyelocyte stage. The molecular basis behind APL has been largely focused on the role of the PML-RARA fusion protein, which interferes with gene expression of hematopoietic progenitor self-renewal as well as myeloid differentiation [26]. CK2 is highly expressed and active in the cytoplasm of APL cells and relocalizes in perinuclear areas upon retinoic acid stimulation. In these cells, CK2 has been found responsible for G1 arrest and a significant amount of the major phosphorylation changes [27].

### 2.7. Adrenocortical Cancer 

Adrenocortical cancer (ACC) is a rare endocrine tumour with a poor prognosis. Current nonsurgical treatment options include radiotherapy and cytotoxic chemotherapy, but margin-negative resection remains the only approach for a durable cure in most cases [28]. CK2 activity has been implicated in human ACC endocrine activity and growth, being an important constituent of a neoplastic milieu [29].

### 2.8. Colorectal Cancer 

Colorectal cancer is the third most common cancer, and its incidence increases with increasing age. Most colorectal cancers are localized with lymph node metastases, and 20% of patients present with metastatic disease, most commonly to the liver. Surgery, radiation therapy, and chemotherapy are the key components of rectal cancer therapy. Studies have shown that patients with recurrent and metastatic disease can be salvaged with surgery and chemotherapy and that substantial progress has been observed in the treatment of metastatic colorectal cancer in recent years [30]. CK2 activity and expression levels are elevated in colorectal tumours, including adenomas and carcinomas [31,32].

### 2.9. Breast Cancer

Breast cancer is one of the most common cancers in women and can commonly transfer to distant organs such as the bone, liver, lung and brain, which mainly accounts for its incurability, although early diagnosis of the disease can lead to a good prognosis. There are numerous risk factors such as sex, aging, and oestrogen gene mutations. Breast tumours usually start from ductal hyperproliferation and then develop into benign tumours or even metastatic carcinomas [33]. High levels of CK2 activity have been detected in breast cancer, where they seem to be necessary to maintain the cancer phenotype [34].

### 2.10. Cholangiocarcinoma 

Cholangiocarcinoma (CCA) is an epithelial cell malignancy arising from varying locations within the biliary tree showing markers of cholangiocyte differentiation. The classification based on anatomical location includes intrahepatic, perihilar, and distal cholangiocarcinoma. Surgery and curative liver transplantation are options for selected patients with perihilar cholangiocarcinoma. However, 5-year survival rates are very low. The chemotherapy regimen of gemcitabine and cisplatin is often used for inoperable disease [35]. In CCA, high CK2 expression is associated with higher tumour grade and impaired survival [36].

### 2.11. Human Cervical Cancer 

Cervical cancer (CC) is the fourth most common cancer among women globally and the fourth most common cause of cancer-related deaths in women. The most important risk factor for the development of CC is cervical infection with human papilloma virus (HPV) [37]. Human cervical cancer invariably demonstrates CK2 transcript upregulation associated with poorer patient survival [38].

## 3. CK2 Structure and Function

**Figure 1:**
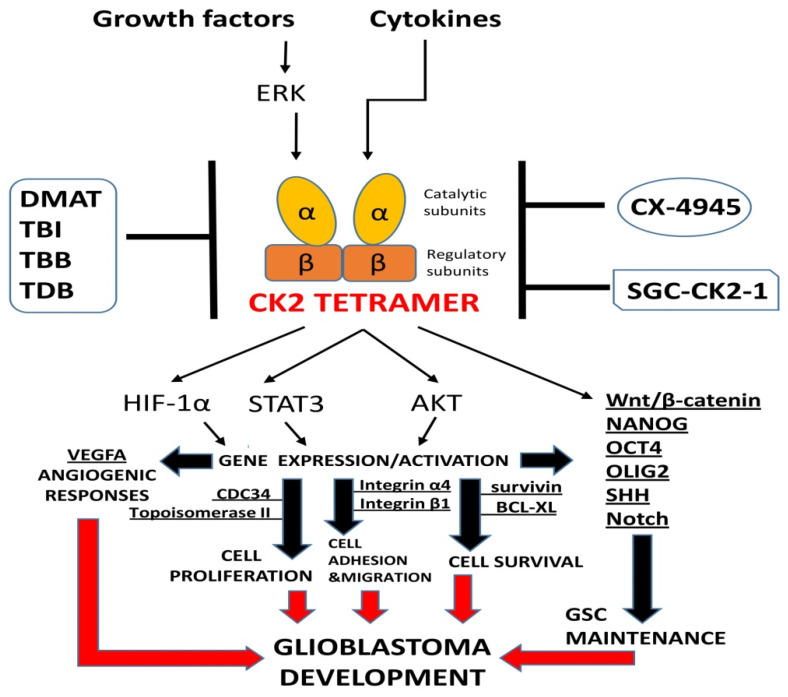
CK2 implications in glioblastoma development. CK2 activity can be stimulated indirectly by growth factors, kinase ERK, and cytokines. Downstream from growth factor signalling, kinase ERK phosphorylates and activates CK2. Activated CK2 regulates AKT and STAT3, HIF-1α, and GSC maintenance to promote glioblastoma cell adhesion, migration, proliferation, and survival. The action of CK2 may lead, through HIF-1α and its targets genes VEGF and MMP2 activation, to angiogenic responses and increased invasiveness [59]. Integrin α4 and Integrin β1 are responsible for glial tumour cell adhesion [60], while CK2-phosphorylated AKT underlies cell protection, as does survivin, the expression of which can be enhanced by CK2 [61,62]. In order to reach this goal, the activation of the antiapoptotic protein BCL-XL is also controlled by CK2 [63]. CDC34 and topoisomerase II are involved in functioning of the cell cycle in glioma cells. CK2 was shown to phosphorylate these cell cycle regulators [51,64]. Several proteins controlled by CK2 have been established to be involved in the maintenance of glioma stem cells, including Wnt/β-catenin, NANOG, OCT4, OLIG2, SHH, and Notch. CK2 is responsible for the phosphorylation of α-catenin and transactivation of β-catenin [65]. The β-catenin-regulated genes OCT4 and NANOG showed significant reductions in expression upon CK2 silencing or pharmacological inhibition in glioma cells [66]. CK2 may also activate SHH and Notch, which are involved not only in stemness maintenance but in mediating chemoresistance (e.g., to TMZ) [67,68]. CK2 regulates gliomagenic functions of Olig2 by participating in phosphorylation of triple serine motif in the amino terminus [69]. Recently, it came under scrutiny whether the use of novel CK2 inhibitors with improved selectivity (e.g., SGC-CK2-1) translated into anticancer effect. DMAT (dimethylamino-4,5,6,7-1H-tetrabromobenzimidazole), TBI (4,5,6,7-tetrabromo-1H-benzimidazol), TBB (4,5,6,7-tetrabromo-1H-benzotriazole), TDB (1-β-D-2′-deoxyribofuranosyl-4,5,6,7-tetrabromo-1H-benzimidazole), CX-4945 (silmitasertib; 5-((3-Chlorophenyl)amino)benzo[c][2,6]naphthyridine-8-carboxylic acid), SGC-CK2-1 (N-(5-(3-Cyano-7-(cyclopropylamino)pyrazolo [1,5-a]pyrimidin-5-ylamino) -2-methylphenyl)propionamide), BCL-XL (B-cell lymphoma-extra large); CDC34 (cell division cycle 34), ERK (extracellular signal-regulated kinase), GSC (glioblastoma stem cells), NANOG (NANOG homeobox), HIF-1α (hypoxia-inducible factor 1α), NF-κB (nuclear factor kappa-light-chain-enhancer of activated B cells), OCT4 (octamer-binding transcription factor 4), OLIG2 (oligodendrocyte transcription factor 2), SHH (Sonic hedgehog), STAT3 (signal transducer and activator of transcription 3), VEGFA (vascular endothelial growth factor A).

CK2 was discovered in 1954 by Burnett and Kennedy. This kinase was isolated from an extract of rat liver, and it was identified with the use of a substrate protein—casein. Hence, it is called CK2 (casein kinase II) [39]. The CK2 holoenzyme is a tetramer comprising two catalytic α- or α’- and two noncatalytic β-subunits. In addition, the two CK2α subunits could be identical (i.e., two CK2α or two CK2α’) or nonidentical (i.e., one CK2α and one CK2α’) [40]. The α-subunits are encoded by two distinct homologous genes: *CSNK2A1*, which encodes CK2α, and *CSNK2A2*, which encodes CK2α’. The β-subunit is encoded by *CSNK2B*. CK2β is not a simple on–off regulator of the catalytic activity of CK2α. It regulates thermostability, substrate specificity, and the ability to attach and penetrate cell membranes [41]. CK2 is constitutively active, and the phosphate group donor is ATP and GTP. CK2 is a pleiotropic kinase that catalyses the phosphorylation of numerous cellular substrates. Many of these proteins are involved in apoptotic signalling pathways. Thus, CK2 is involved in a complex series of cellular functions, including maintaining cell viability. CK2 can exert an antiapoptotic role by protecting regulatory proteins from caspase-mediated degradation, and this antiapoptotic function of CK2 may contribute to its ability to participate in tumorigenesis [40]. High levels of CK2 have been reported in many neoplasms [42], including neoplasms of the central nervous system such as glioblastoma [43,44,45,46] and medulloblastoma [18,47]. Extensive research has shown that in glioblastoma, CK2 regulates many cell signalling pathways and processes including proliferation, rRNA and tRNA synthesis, apoptosis, the cell cycle, and DNA damage [11,17,48] (Figure 1). Additionally, it activates signalling pathways, e.g., JAK/STAT, NF-κB, PI3K/Akt, and regulates suppressor proteins PTEN and p53 and proto-oncogenes c-Myc and c-Myb [11]. It participates in the protection of antiapoptotic proteins [49] and shows proangiogenic activity [34]. 

Numerous studies show that the CK2α and CK2β subunits are subjected to different physical forces that lead to the reversible formation of different molecular forms, e.g., the tetrameric holoenzyme. This makes it possible to target the surface of each kinase subassembly by its small molecule inhibitors (Appendix A). Most inhibitors target the ATP binding pocket using hydrogen bonding and hydrophobic interactions [3]. In cells, CK2α and CK2α’ were identified as bona fide targets of TBB (4,5,6,7-tetrabromo-1H-benzotriazole), TBBz (4,5,6,7-tetrabromo-1H-benzimidazole), and DMAT (2-dimethylamino-4,5,6,7-1H-tetrabromobenzimidazole). The binding site for CK2 inhibitors in this hydrophobic pocket is located at the interface with CK2β [50,51]. Lowering the hyperactivity of CK2 by chemical or molecular methods induces apoptosis in cells and has a significant effect on the inhibition of tumorigenesis [52,53]. Research into compounds that are kinase inhibitors has been going on for several decades, including research into CK2 inhibitors [54]. The class of CK2 inhibitors (competitive inhibitors) directed to the active site with ATP includes, among others, compounds such as 4,5,6,7-tetrabromobenzimidazole (TBB) derivatives [55], polyphenol derivatives [56], and indolequinazoline derivatives [57]. These compounds show high specificity for CK2 and show high efficacy in the low micromolar range [49]. The benzimidazole derivative family began with a scaffold derived from the 5,6-dichloro-1- (β-d-ribofuranosyl) benzimidazole (DRB) molecule. Based on this, the structure of the inhibitors was optimized to better fit the ATP binding pocket. Therefore, the most effective inhibitors of CK2, which are also derivatives of benzimidazole, are TBB, TBI (4,5,6,7-tetrabromo-1H-benzimidazole), and DMAT compounds. In general, benzimidazole derivatives have effectively inhibited both native and recombinant CK2 activity in in vitro tests and shown proapoptotic properties on various tumour lines [58].

## 4. DRB/DRB Derivatives CX-4945 and ZKK

### 4.1. DRB

**Figure 2:**
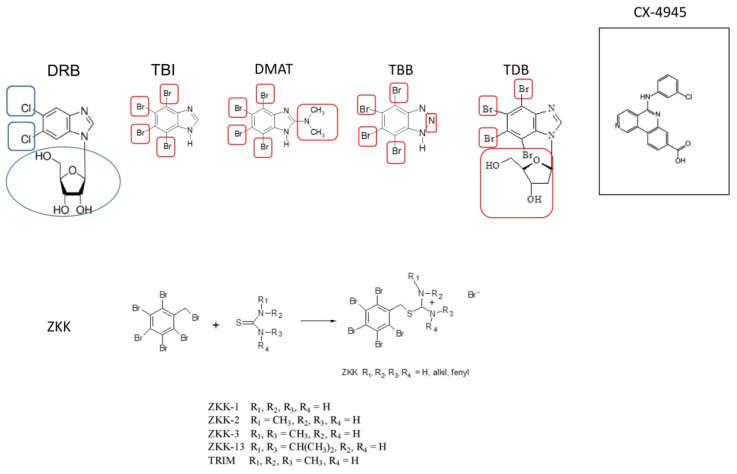
Chemical structure of DRB and its derivatives, CX-4945 and ZKK, with encircled modifications of DRB structure. Modifications that nucleoside DRB underwent included the deletion its sugar moiety and the replacement of the chlorine and hydrogen atoms of its benzene ring. Encircled among others are bromine atoms that are critical for encapsulating inhibitors in the hydrophobic cavity of CK2. The bottom panel shows the chemical synthesis of ZKK.

The CK2 protein kinase inhibitor 5,6-dichloro-1-β-D-ribofuranosyl-1H-benzimidazole (DRB) was described by Zandomeni et al. in 1986 [70]. It is still employed as a CK2 inhibitor despite its low efficacy (IC_50_ around 15 μM) [71]. DRB reduced glioma cell viability in vitro, inhibited TNFα (tumour necrosis factor α)-mediated NF-κB activation, and sensitized cells to TNFα-induced apoptosis [17]. Because of its weak inhibitory properties, attempts were made to modify it. To this end, the sugar part was removed from the compound molecule, and the chlorine derivatives of benzimidazole were replaced with bromine derivatives. Four added bromine atoms were critical to encapsulate the inhibitor in a relatively small hydrophobic cavity. Therefore, selectivity toward CK2 was strengthened. In addition, the negative charge that is present on the triazole ring of TBB, and not present in DMAT or TBI, makes TBB less effective on kinases other than CK2 [72]. Through various modifications of the 5,6-dichloro-1-β-D-ribofuranosyl-1H-benzimidazole (DRB) molecule, new tetrabromo-benzimidazole and benzotriazole inhibitors were developed [71]. The summary of the chemical structure of DRB and its derivatives, CX-4945 and ZKK, is presented in the Figure 2.

### 4.2. TBI

An example of tetrabromo-benzimidazoles would be 4,5,6,7-tetrabromo-1H-benzimidazole TBI (TBBz or TBBi) (TBI IC_50_ = 0.5 μM) [50,72]. It has been demonstrated that the compounds TBI and DMAT (the latter also made from the modification of DRB) are potent inhibitors of PIM (proviral insertion site in Moloney murine leukaemia virus) family kinases, including PIM2 and PIM3, and of other kinases, for example, PKD1 (protein kinase D1), HIPK2 (homeodomain-interacting protein kinase 2), and DYRK1a (dual-specificity tyrosine phosphorylated and regulated kinase 1a) [72].

In a study by Pucko et al., it was observed that T98G cells, after 24 and 48 h of incubation with 4,5,6,7-tetrabromo-1H-benzimidazol (TBI) at concentrations of 25–100 µM, showed statistically significant changes in cell viability and proliferation, with T98G control without compounds as reference. After 48 h of incubation, TBI decreased the number of SEGA (subependymal giant cell astrocytoma) cells (SEGA is a benign brain tumour of childhood) at concentrations of 25–100 µM [73]. The cytotoxic effect of TBI was also documented in other studies, where after 24 h incubation, TBI at 50 µM resulted in a reduction in cell viability in rat glioma C6 cells. TBI at higher concentration was very effective in the induction of cell death in T98G cells [74]. Most tumours contain mutations in the genes encoding kinases, including kinases that are parts of important signalling pathways such as the PI3K/Akt/mTOR pathway. Studies have shown that various CK2 inhibitors, including TBI, may also participate in the modulation and transduction of many signalling pathways, including mTOR kinase-related pathways, which play a dominant role in the formation of SEGA tumours [73]. However, further studies are needed to elucidate the exact mechanism of treatment.

### 4.3. DMAT

The compound 4,5,6,7-tetrabromo-1H-benzimidazole-2-N, N-dimethylamine (DMAT) (another record: 2-dimethylamino-4,5,6,7-1H-tetrabromobenzimidazole) was also made from the modification of DRB [55]. DMAT, with IC_50_ = 0.14 μM (as determined in one study), has a remarkable affinity and selectivity for CK2 [50]. This compound easily penetrates into cells. Additionally, it has the lowest kinetic Ki value among CK2 inhibitors (Ki = 0.040 µM). Studies have shown that DMAT exerts a better antitumour effect than TBB. This compound is also more effective at inhibiting CK2 [75]. Studies on malignant glioma cells of the T98G lineage showed that DMAT reduced viability and proliferation [73]. It also caused a decrease in cell viability in the range of 10–50 µM after 24 h of incubation with the LN229 glioma cell line, while in T98G cells, it induced the activation of caspases 3, 7, and 8; increased the expression of FasL and Fas; and weakened the membrane potential and mitochondrial function [74]. Other reports have also indicated high effectiveness of this compound. For example, in malignant lymphoblastic leukaemia cells, it inhibited growth with better efficiency than imatinib [76,77]. It shows cytotoxic activity in the range of 20–40 µM in in vitro cells of colorectal cancer and breast cancer [78]. It induced apoptosis of human MCF-7 breast cancer cells at a concentration of 10 µM, as well as death by apoptosis in human acute leukaemia myeloid cells (KG-1), and when in combination with pentabromobenzylisothiouronium bromides (ZKK-13), it showed a synergistic proapoptotic effect [79]. On the other hand, a significant inhibitory effect on the viability of human adrenocortical cancer cell line (H295R) has been documented after 72 h incubation with DMAT at concentrations 4–10 µM [80].

### 4.4. TBB

TBB (or TBBt), 4,5,6,7-tetrabromo-1H-benzotriazole is a benzotriazole derivative IC_50_ 0.5 µM [50]. In 1995, the compound was reported as a potent and selective inhibitor of CK2 [81]. The structure of this compound was based on the backbone of a known DRB inhibitor [50]. The newly formed TBB compound is characterized by a low Ki value (Ki = 0.4 µM) and an ATP site-directed protein kinase inhibitor, and it perfectly fits and fills the hydrophobic CK2 pocket [82]. Research by E. Pucko et al. has documented that TBB, at 75 μM and 100 µM concentrations after 24 h and 48 h of incubation, reduced the proliferation of T98G malignant glioma cells. Studies have shown that TBB has a lower cytotoxic efficacy against glioblastoma cells than TBI and DMAT [73,74]. TBB inhibits PIM family kinases including PIM1 and PIM3 as well; however, the highest selectivity is for CK2 [72]. In addition, studies in human stromal cells in chronic lymphocytic leukaemia (CLL) showed that TBB inhibited CK2 and induced time- and dose-dependent cell death by apoptosis [57,82], which was accompanied by a reduction in PTEN and Akt phosphorylation [83].

### 4.5. TDB

The TBI inhibitor was the basis for the development of another CK2 inhibitor compound. By modifying the TBI molecule by adding deoxyribose, 1-β-D-2′-deoxyribofuranosyl-4,5,6,7-tetrabromo-1H-benzimidazole K164 (also known as TDB) was formed (IC_50_ for CK2 of TDB = 32 nM) [84]. TDB benzimidazole belongs to the group of ATP competitors and inhibitors of kinases CK2, PIM1, CLK2, and DYRK1A. One study showed that TDB readily permeated cells and induced apoptosis of neoplastic cells [85]. CK2 inhibitors have been proven to pass the BBB; however, the exact mechanism of passage through the BBB is far from being understood and thus requires further studies. Even more importantly, CK2 can regulate the activity of multidrug resistance pumps. It has been revealed that CK2 phosphorylates and upregulates the P-glycoprotein (P-gp, also known as ABCB1), a product of the multidrug resistance 1 (MDR1) [86]. Therefore, inhibitors can be used as “boosters” to overcome the MDR phenomenon and increase the uptake of chemotherapeutics. The cell permeability of CK2 inhibitors has been experimentally confirmed by demonstrating an inhibited endogenous CK2 in cell lysates and a depletion of phosphosites directly generated by CK2 [85]. In vitro studies have shown that TDB reduces proliferation of glioblastoma cells and that coadministering hyperbaric oxygen (HBO) potentiates the action of this compound (Pucko et al., unpublished data). A study by G. Cozza on the CEM (human T-lymphoblastoid cells) and HeLa (human cervical cancer cells) cell lines showed a significant decrease in cell viability. The cytotoxic/antiproliferative effect of TDB on CEM cells was almost entirely due to apoptosis, whereas percent necrosis was very small [85]. Nevertheless, importantly, TBB, DMAT, and TBI as glioblastoma treatments tend to relatively selectively target glioblastoma cells, while normal cellular components of the brain are moderately resistant to their action [73] (Pucko 2021, unpublished observation). However, in order to further substantiate this notion, indexes of selectivity should be evaluated in further studies.

### 4.6. CX-4945

Other research has shown that the inhibitor CX-4945 is also very effective in inducing apoptosis and cell death. It needs to be highlighted that among the compounds reviewed here, CX-4945 is the only molecule of which the chemical structure is not derived from that of DRB. It has been determined that the binding pocket of CK2α is composed of hydrophobic regions, a positive area, and a hinge region. CX-4945, an inhibitor with high inhibitory activity (IC_50_ = 0.3 nM), establishes interactions with the hinge and positive regions via its pyridine and carboxylate groups, respectively. The tricyclic skeleton of CX-4945 assures strong contacts with residues in the hydrophobic regions, thus stabilizing binding to CK2 [87]. The first oral small-molecule CK2 inhibitor is 5- (3-chlorophenylamino) benzo [c] [2,6] naphthyridine-8-carboxylic acid (CX-4945), the activity of which has been assessed in vitro and in vivo [32,88]. To date, many inhibitors of CK2 have been described in the literature, but CX-4945 (silmitasertib) was the first compound to enter clinical trials (NCT00891280, NCT02128282) and to be effective in both human haematological and solid tumours. In addition, CX-4945 has the ability to synergistically work with various classes of anticancer agents, which can establish multidirectional approaches to cancer [89]. It has been proven that CX-4945 may act synergistically with several anticancer drugs such as gemcitabine, cisplatin, and bortezomib against cholangiocarcinoma and acute lymphoblastic leukaemia [90,91]. Studies have shown that CK2 is involved in inducing medulloblastoma tumorigenesis. However, CX-4945 inhibited the proliferation of different medulloblastoma cell lines, while CX-4945 treatment in association with temozolomide strongly delayed cell growth and promoted apoptosis in vitro, thus showing a strong synergy between both drugs [18]. CX-4945, when administered with gefitinib, an epidermal growth factor receptor (EGFR) inhibitor, exerted a strong antiproliferative effect on glioblastoma in vitro [45]. Genetic EGFR alterations have been found in about 60% of glioblastoma patients, leading to uncontrolled activation of signalling pathways (MAPK, PI3K/AKT, JAK/STAT, NF-κB, AKT, and others), which in turn promote cell and tumour growth, apoptosis resistance, and angiogenesis. However, EGFR-targeted therapies brought poor results in patients with glioblastoma [92,93].

For quite some time, CX-4945 was referred to as a compound with a relatively high selectivity towards CK2. CX-4945 was found to be selective for CK2 when evaluated in a 235-kinase biochemical panel [72,94]. However, a newly developed compound, SGC-CK2-1, which belongs to the pyrazolo-pyrimidines, was synthesized via acylation of the aniline followed by reduction of the nitro group and coupling with the pyrazolo-pyrimidine core [95]. SGC-CK2-1 showed stronger inhibition of both CK2 catalytic subunits (IC_50_ = 36 nM and 16 nM for CK2α and CK2α’ HEK-293 cells, respectively) than CX-4945 (IC_50_ of 45 nM for CK2α’). In a panel of 403 kinases, CX-4945 inhibited 28 kinases, while SGC-CK2-1 inhibited 3 kinases (including CK2α and CK2α’), by >90% at 1 µM. This pointed towards much higher selectivity of SGC-CK2-1 regarding CK2 inhibition. However, SGC-CK2-1 showed neither antiproliferative activity against U-87 MG cells nor caspase 3/7 activation. This gave rise to the notion that the antiproliferative activity exhibited by less selective CK2 inhibitors was due to off-target effects. Nevertheless, it seems that any reliable conclusions should be withdrawn at this point, considering that the investigation is still ongoing. In addition, recent reports seem to have indicated that other inhibitors under development that are more specific than CX-4945 showed anticancer effect [96].

Modification of a biologically active molecule by introducing various substituents may result in an unexpected effect. For example, the addition of chlorine in the structure of a compound may result in its low or no cytotoxic activity, as in the case of BEN compound, which showed very little or no cytotoxicity towards low- (G1) and high-grade (G4) glioma cells [97]. TBB, TBI, and DMAT compounds show strong CK2 inhibitory properties, while S-pentabromobenzylisothiourea derivatives, which are structurally similar to polybrominated compounds (TBB, TBI, DMAT), show a distinct protein kinase inhibition profile. Isothioureas are a class of amphiphilic compounds with very basic functions of isothiourea, with pKa ≈ 10. Under physiological pH conditions, these compounds exist in a proton form, which may be of importance for their specific effects in the cell. The synthesis of these compounds is not demanding, because they show poor solubility in the reaction medium. In the solid form, they form salts, usually with better solubility in water, which makes them particularly attractive compounds for scientific research [79,98].

Isothioureas are also blockers of CXCR4 receptors, which, when combined with the CXCL12 chemokine (a.k.a. stromal cell-derived factor 1, SDF-1), activate various signalling pathways, including phosphatidylinositol kinase-3 (PI3K)/AKT. Subsequently, other signal transduction pathways are triggered, including the MEK/MAP pathway, which is associated with the proliferation and survival of tumour cells [99,100]. It has been shown that glioblastomas exhibit the highest levels of expression of the SDF-1 chemokine and the CXCR4 receptor, which makes these tumours even more virulent [101].

### 4.7. ZKKs

One of the pentabromobenzylisothiourea compounds, N, N’-dimethyl-S-2,3,4,5,6-pentabromobenzylisothiourea (ZKK-3), at a concentration of 10 µM, inhibited the activity of protein kinases to a different level, expressed as the residual activity (i.e., percentage of the control activity without inhibitor), but did not inhibit CK2, as determined with kinase profiling assay methods at the Division of Signal Transduction Therapy, University of Dundee [98]. 

The isothiourea derivatives ZKK-1, ZKK-2, ZKK-3, ZKK-4 and ZKK-5 (ZKKs) showed cytotoxic and proapoptotic activity in the HL-60 line (human promyelocytic leukaemia) and in the K-562 line (human chronic erythromyeloblastic leukaemia) [79]. It has also been shown that ZKKs (ZKK1–8, IC_50_, 7–50 μM) have a cytotoxic effect on glioblastoma cells, including the isothiourea derivative ZKK-1 showing an inhibitory effect on the survival of C6 rat glioma cells and human glioblastoma lines (LN229 and T98G) in vitro [74]. Extended studies on isothiourea derivatives showed that these compounds, including the S-pentabromobenzylisothioureas derivatives S-(2,3,4,5,6-pentabromobenzyl) -isothiouronium bromide (ZKK-1), N-methyl- S-(2,3,4,5,6-pentabromobenzyl) -isothiouronium bromide (ZKK-2), N,N’-dimethyl- S-(2,3,4,5,6-pentabromobenzyl) -isothiouronium bromide (ZKK-3), N,N’-diisopropyl- S-(2,3,4,5,6-pentabromobenzyl) -isothiouronium bromide (ZKK-13), and N,N,N’-trimethyl- S-(2,3,4,5,6-pentabromobenzyl)-isothiouronium bromide (TRIM), had various cytotoxic and proapoptotic effects on glioblastoma cells [97]. 

Additionally, studies have shown that the combination of HBO with the ZKK3 isothiourea derivative increases cytotoxicity and makes T98G cells more sensitive to antitumour effect [102]. Thus, HBO may promote sensitivity to molecular targeted therapy in glioblastoma cells. The question of whether the mechanism relies on potentiating kinase inhibitory effects or suppression of hypoxia inducible factor 1α (HIF-1α)- and HIF-2α-dependent mechanisms requires further studies [103]. HBO therapy is a treatment that delivers 100% oxygen at a pressure greater than atmospheric pressure at sea level. Research in recent years has shown that these compounds can be suitable as components of combined therapies with HBO. Such a combination increases the susceptibility of glioma tumour cells to cell death via apoptosis. The possible synergy of CK2 inhibitors and HBO could be based on HBO targeting hypoxic signalling and therefore diminishing hypoxia-driven CK2 intratumoural expression alongside the suppression of CK2 activity by inhibitors [104]. HBO the improved efficacy of anticancer drugs and may help improve oxygen tension within the hypoxic regions of the neoplastic tissue [105]. Thus, patients undergoing treatment with these compounds might receive oxygen therapy in a hyperbaric chamber. However, further investigations are needed to establish HBO as an adjuvant treatment to potentiate radio- and chemotherapy treatment of gliomas.

Extended studies of ZKK3’s properties showed that it inhibits about 70 percent of the activity of seven kinases, ERK8, PKD1, NEK2a (never in mitosis (NIMA)-related kinase 2a), PIM1, PIM3, IGF-1R (insulin-like growth factor-1 receptor), and IR (insulin receptor), that play an important role in the invasiveness of gliomas [98]. These kinases inhibited by ZKK3 include, but are not limited to, PIM kinases. PIM kinases also play a crucial role in glioma cell signalling pathways [106] and are involved in the regulation of cancer stem cells (e.g., PIM-3 kinase is overexpressed in glioblastoma stem cells) [107]. Various studies have shown that the inhibition of PIM3 kinase activity induces apoptosis and a suppression of glioblastoma cell proliferation [108]. Concordantly, overexpression of PIM kinases correlates with a poor prognosis in the treatment of neoplasms, including glioblastoma. ZKK-3 also inhibits insulin-like growth factor-1 receptor (IGF-1R) and insulin receptor (IR) kinases overexpressed by glioblastoma [79,109]. The insulin receptor (IR) belongs to the receptor tyrosine kinases and has two isoforms, IR-A and IR-B, which differ in the structure of the α-subunit and in ligand-binding capacity. IR binds insulin and regulates cellular metabolism by activating the PI3K/AKT pathway [110]. The inhibition of IR reduces proliferation and increases the sensitivity of cancer cells to anti-IGF-1R therapies [111]. Another kinase inhibited by ZKK3 is PKD1, which belongs to the PKD family of kinases [97]. The kinases of this family play various roles in biological processes including cell metabolism. The state of knowledge on the expression and function of PKD in gliomas is limited, although recent studies have shown that ZKK3 inhibits the activity of PKD1 in glial cell lines [97] as well as that of PKD isoforms under various tumour oxygen conditions [102]. 

## 5. Other Inhibitors of Kinases

### 5.1. EGFR Inhibitors

Inhibitors of other kinases, potentially combined with CK2 inhibitors, may prove instrumental in developing clinically successful therapies for patients with gliomas. The family of RTK catalytic receptors, which regulates various biological processes, is responsible for the activation of many signalling pathways in the cell [112]. As a result of genetic changes in the cell, RTK is deregulated [113]. Epidermal growth factor receptor (EGFR) signalling leads to the activation of the MAPK pathway, as well as the PI3K pathway and other pathways intracellularly. Overexpressed EGFR, which was seen in 22–89% of glioblastomas [114,115], disrupts downstream signalling pathways, including PI3K, Akt, and MAPK. Attempts to inhibit EGFR, or the mutant form EGFRvIII, using the biological drugs cetuximab, panitumumab, and nimotuzumab have not been successful [116,117]. Likewise, though gefitinib, erlotinib, and afatinib inhibited EGFR in vitro, reduced proliferation and angiogenesis in glioblastoma cells, these results were not confirmed in the clinic [118]. However, the third-generation EGFR inhibitor osimertinib (AZD9291), which crosses the BBB and inhibits the proliferation of glioblastoma cells, is very promising [119].

### 5.2. PI3K/Akt/mTOR Inhibitors

Binding to receptor tyrosine kinases activates the PI3K/Akt/mTOR pathway. Its first member, which is phosphatidylinositol 3-kinase (PI3K), belongs to the lipid kinase family and is often hyperactive in glioblastomas. PI3K catalyses the conversion of phospatidylinositol-4,5-diphosphate (PIP2) to phosphatidylinositol-3,4,5-triphosphate (PIP3), which is regulated by PTEN (phosphatase and tensin homolog). However, PTEN mutation is a genetic feature in 50% of patients with glioblastoma. The loss of PTEN function results in a great activation of AKT kinases and mTOR kinases. The permanently activated RTK/PI3K/Akt signalling path in neoplastic cells promotes the development of neoplasms, including gliomas. Therefore, it is important to develop inhibitors to counteract these molecular events. Among many AKT kinase inhibitors is buparlisib (BKM120), which induces apoptosis and G2/M cell cycle arrest in glioblastoma cells, prevents the growth of intracerebral U87 MG glioblastoma xenografts, and prolongs survival, as determined in preclinical studies [120]. Interestingly, BKM120 passes the BBB and has been used in combination with other drugs or radiotherapy and in clinical trials for glioblastoma [121].

mTOR kinases include mTORC1 and mTORC2, which are made of, among others, p70S6K1 kinase, playing an important role in the formation of malignant gliomas. TORC2, on the other hand, activates Akt and PKCα. All kinases play important roles in proliferation, survival, and procellular regulation. In contrast, breaking their action may provide an anticancer effect. mTORC1 inhibitors include rapamycin (sirolimus) and its analogues, such as RAD001 (everolimus), CCL-779 (temsirolimus), and AP23573 (ridaforolimus) [122]. The AZD8055 inhibitor reduces the growth and activity of S6 and AKT kinases in vivo [123]. On the other hand, the Torin1 inhibitor inhibits the activity of the mTORC1 and mTORC2 complex and is well compatible with TMZ in glioblastoma cells [124]. Synergy between Torin1 and rapamycin has also been shown to inhibit cell migration and interfere with the Wnt/β-catenin pathway in glioblastoma cells [125]. Additionally, the combination of Torin1 and AZD8055 mediates the internalization of EGFR [124]. Research has shown that PI3K/Akt affects Bcl-2 proteins and, more specifically, the expression levels of pro- and antiapoptotic proteins. The level of Bcl-2 proteins can determine the fate of cancer cells—its reduction may increase susceptibility to chemotherapy. To this end, it has been shown that double inhibitors, e.g., dactolisib (NVP-BEZ235), which inhibits PI3K/mTORC1/2 and reduces the activation of AKT kinase, give improved results. It has been proven that dactolisib participates in the induction of apoptosis by increasing the expression of the proapoptotic proteins Bax and caspase-3 and sensitizes glioblastoma cells to radiotherapy in vivo [126]. This compound (NVP-BEZ235) was included in a Phase IIB study (NCT02430363), in combination with pembrolizumab (MK-3475, monoclonal antibodies), on the treatment of glioblastoma patients [121,127]. GDC-0084 (RG7666) is a PI3K/mTOR inhibitor capable of crossing the BBB. Based upon the results of in vitro studies, it inhibited the proliferation and growth of U87 MG glioblastomas, possibly by reducing the phosphorylation of Akt kinase [128].

### 5.3. Therapies Combined with CK2 Inhibitors

As mentioned before, these therapeutics agents targeting other kinases can be potentially combined with CK2 inhibitors, as demonstrated by several authors [89,90,91]. Several authors have so far provided interesting insights on how CK2 inhibitors behave with these compounds in combination therapy. In a study by Bliesath et al., EGFR and CK2 were inhibited with a combination of CX-4945 and erlotinib (i.e., EGFR tyrosine kinase inhibitor) in in vitro models of cancer (non-small cell lung carcinoma, squamous cell carcinoma cells). This combination enhanced attenuation of the PI3K/Akt/mTOR pathway more than EGFR inhibitor alone, including enhancing the tumour cell killing effect, which is unsurprising given the positive role of CK2 in phosphorylating prosurvival Akt [129]. The CK2 inhibitor CX-4945 was also combined with selumetinib, an inhibitor of mitogen-activated protein kinase 1/2 (MEK 1/2), to treat non-small cell lung cancers, synergizing in targeting cancer cell survival, proliferation, differentiation, and migration [130]. Other classes of kinase inhibitors that CX-4945 cooperates with are GS-1101 (idelalisib), a phosphoinositide 3-kinase p110δ (PI3Kδ) inhibitor; ibrutinib, a potent and irreversible inhibitor of Burton’s tyrosine kinase (BTK); imatinib, for treatment of haematological malignancies; dasatinib (an inhibitor of Src family tyrosine kinases), for treatment of ovarian cancer; and LY2157299 (a TGF-β receptor I kinase inhibitor), for treatment of human cholangiocarcinoma [89,131].

Combined treatments have been also proposed for glioma. As mentioned before, CX-4945, combined with gefitinib (an EGFR inhibitor), exerted a strong antiviability effect on glioblastoma cells in vitro [45]. Therefore, further studies utilizing this approach are warranted, possibly including BBB modulators in order to increase levels of kinase inhibitors in glioma tissues in vivo [132].

Several compounds other than kinase inhibitors have also shown suitability to be combined with CX-4945, including gemcitabine and cisplatin, for treatment of cholangiocarcinoma cells and grafts [91]. The proteasome inhibitor bortezomib was combined with CX-4945 to experimentally treat acute lymphoblastic leukaemia. A synergistic apoptotic effect was observed, as BIP/Grp78—ER chaperone, as well as the antiapoptotic genes BCL-XL and XIAP, were profoundly repressed under the combined treatment [90]. Importantly, CX-4945 potentiated the antiglioma effect of temozolomide by reducing the function of CK2-dependent O-6-methylguanine-DNA methyltransferase (MGMT) [133].

Among frequently reported effects of combined therapies are the significant enhancement of signalling pathway interference, oftentimes leading to apoptosis, and the impairment of neoplastic cell growth. However, data have also shown that certain molecular events working towards killing neoplastic cells are obtainable only when combination treatment with inhibitors is applied. CK2 inhibition may synergize with other kinase inhibitors and sensitize to pharmacological (e.g., TMZ) and nonpharmacological (e.g., thermal stress or HBO) treatments [102,134]. 

## 6. Effects of Silencing CK2 on Glioma Development

The inhibition of CK2 is not limited to the described inhibitors, as other methods targeting CK2 have been used in researching the role of CK2 in major pathways of glioma development, including cell proliferation, adhesion and migration, survivability, and stemness maintenance, thus carrying therapeutic potential. Small interfering RNA for CK2 suppressed activation of the JAK/STAT, NF-κB, and AKT pathways and downstream gene expression in human glioblastoma xenografts as well as decreasing U251-MG cell growth [46]. CK2 siRNA reduced glioma cell viability, inhibited TNFα-mediated NF-κB activation, and sensitized cells to TNFα-induced apoptosis [17]. In vivo study further verified the validity of targeting CK2 with siRNA, which reduced cell growth, decreased tumour size, and increased survival rates in GBM xenograft mouse models [66]. In this study, inducible short hairpin RNAs (shRNAs) specific to CK2α resulted in reductions in markers of stemness and the sphere-forming capacity of brain tumour-initiating cells, thus confirming the importance of CK2α in glioblastoma stem cell maintenance. This was also confirmed by siRNA knockdown of the CK2 catalytic subunits, which reduced neurosphere formation in glioblastoma xenolines [45]. Reducing the expression of CK2 subunits with siRNA resulted in a decreased proliferation, survival, migration, and invasiveness in malignant glioma cells and a variety of other cancer cells. Knockout of CK2 with the use of CRISPR technology further confirmed reduced cell proliferation, motility, and invasiveness as a result of CK2 targeting [96].

## 7. Conclusions

Among the CK2 inhibitors reviewed here, CX-4945 appears particularly interesting for further research, judging from the preclinical data. This inhibitor exerted an antiproliferative effect verified both in vitro and in vivo (human glioblastoma xenografts), which cannot be said for all CK2 inhibitors. In addition, orally administered CX-4945 showed high bioavailability, over 70%, and was well tolerated. CX-4945 could trigger antiangiogenic and anti-inflammatory responses and CK2-dependent HIF-1α transcriptions. In addition, it has been proven as a valuable component of combined therapies, e.g., with gefitinib [32,45,46,135]. The described concentrations of CK2 inhibitors are achievable in vivo. Hence, CK2 inhibitors were administered in several in vivo studies with measurable therapeutic concentrations [42,135]. Research into anticancer therapies is still a huge challenge, and therefore, new methods and chemicals must be sought to combat this disease. The development of CK2 inhibitors resulted in a variety of agents with broadened kinase inhibitory profiles, and the search for compounds with improved selectivity continues. On the other hand, a high selectivity towards CK2 may not result in antitumour effectiveness, which has been associated with earlier inhibitors, possibly because of off-target effects. There is still a paucity of studies investigating the molecular mechanisms of cell penetration and distribution of CK2 inhibitors as well as antiglioma synergic effects with other kinase inhibitors and treatment modalities. The antiglioma effectiveness of novel CK2 inhibitors needs to be further verified in clinical trials. Combined therapies for glioma, with inhibitors of kinases and HBO, have brought promising results in recent years. In this respect, clinical studies are still awaited.

## Data Availability

Not applicable.

## List of Abbreviations

**ABCB1**	P-glycoprotein, also known as ABCB1
**AKT**	protein kinase B
**AP23573**	ridaforolimus
**ATP**	adenosine 5′-triphosphate
**AZD8055**	mTOR inhibitor
**Bax**	Bcl-2-associated protein X
**BBB**	blood–brain -barrier
**BCL-XL**	B-cell lymphoma-extra large
**Bcl-2**	B-cell CLL/lymphoma 2
**BEN**	S-benzylisothiourea hydrochloride
**BRAFV600**	mutation of the B-Raf gene in which valine is substituted by glutamic acid at amino acid 600
**BKM120**	buparlisib
**CCL-779**	temsirolimus
**CDC34**	cell division cycle 34
**CDKN2A/B**	cyclin-dependent kinase inhibitor 2A/B
**CEM**	human T-lymphoblastoid cells
**CK2**	casein kinase 2
**CK2α**	casein kinase 2 alpha
**CK2β**	casein kinase 2 beta
**CLK2**	CDC-like kinase 2
**c-Myc**	myelocytomatosis viral oncogene homolog
**c-Myb**	V-myb avian myeloblastosis viral oncogene homolog
**CNS**	central nervous system
**CSNK2A1**	gene encoding casein kinase 2 alpha 1
**CSNK2A2**	gene encoding casein kinase 2 alpha 2
**CSNK2B**	gene encoding casein kinase 2 beta
**CXCL12/SDF-1**	stromal derived factor-1
**CX-4945**	(silmitasertib) 5-((3-Chlorophenyl)amino)benzo[c][2,6]naphthyridine-8-carboxylic acid)
**NVP-BEZ235**	dactolisib
**DMAT**	2-dimethylamino-4,5,6,7-1H-tetrabromobenzimidazole
**DNA**	deoxyribonucleic acid
**DRB**	5,6-dichloro-1-beta-D-ribofuranosylbenzimidazole
**DYRK1a**	dual-specificity tyrosine phosphorylated and-regulated kinase 1a
**EGFRvIII**	epidermal growth factor receptor variant III
**EGFR**	epidermal growth factor receptor
**ERK**	extracellular signal-regulated kinase
**FDA**	Food and Drug Administration
**FasL**	Fas ligand
**GSC**	glioblastoma stem cells
**GTP**	guanosine-5’-triphosphate
**HBO**	hyperbaric oxygen
**HeLa**	human cervical cancer cells
**HIF-1α**	hypoxia-inducible factor 1α
**HIF-2α**	hypoxia- inducible factor 2α
**HIPK2**	homeodomain-interacting protein kinase 2
**HL-60**	cell line human promyelocytic leukaemia
**H295R**	human adrenocortical cancer cell line
**IDH1/IDH2**	isocitrate dehydrogenase 1/2
**IGF-1R**	insulin-like growth factor-1 receptor
**IR**	insulin receptor
**IR-A**	insulin receptor isoform A
**IR-B**	insulin receptor isoform B
**JAK**	Janus kinase
**K-562**	cell line human chronic erythromyeloblastoid leukaemia
**KG-1**	cell line human acute myelogenous leukaemia
**LN229**	glioma cell line
**MAPK**	mitogen-activated protein kinases
**MCF-7**	breast cancer cells
**MDR1**	multidrug resistance 1
**mTOR**	mammalian target of rapamycin
**mTORC1**	mammalian target of rapamycin complex 1
**mTORC2**	mammalian target of rapamycin complex 2
**NANOG**	NANOG homeobox
**NEK2a**	never in mitosis (NIMA)-related kinase 2a
**NF-κB**	nuclear factor kappa-light-chain-enhancer of activated B cells
**OCT4**	octamer-binding transcription factor 4
**OLIG2**	oligodendrocyte transcription factor 2
**AZD9291**	osimertinib
**MK-3475**	pembrolizumab, monoclonal antibodies
**PI3K**	phosphatidyl-inositol 3-kinase
**PIM**	proviral insertion site in Moloney murine leukaemia virus
**PIM2**	kinase PIM2
**PIM3**	kinase PIM3
**PIP2**	phosptidyl-inositol-4,5-diphosphate
**PIP3**	phosphatidyl-inositol-3,4,5-triphosphate
**PKCα**	protein kinase C alpha
**PKD**	protein kinase D
**PKD1**	protein kinase D1
**PTEN**	phosphatase and tensin homolog deleted on chromosome 10
**pTERT**	telomerase reverse transcriptase promoter
**p53**	tumour protein
**p70S6K1**	ribosomal S6 kinase p70
**RAD001**	everolimus
**RNA**	ribonucleic acid
**rRNA**	ribosomal RNA
**SDF-1**	chemokine and the CXCR4 receptor
**SEGA**	subependymal giant cell astrocytoma
**SGC-CK2-1**	N-(5-(3-Cyano-7-(cyclopropylamino)pyrazolo[1,5-a]pyrimidin-5-ylamino)-2-methylphenyl) propionamide
**SHH**	Sonic hedgehog
**shRNAs**	short hairpin RNAs
**siRNA**	small interfering RNA
**STAT3**	signal transducer and activator of transcription 3
**S6Ks**	S6 kinases

## Appendix A

**Table A1:** Activity and cytotoxicity of selected kinase inhibitors in different cell models.

**Compound**	**Activity and Cytotoxicity on Different Cell Models**	**Possible Off-Target Interactions**	**Reference**
5,6-dichloro-1-β-D-ribofuranosyl-1H-benzimidazole **(DRB)**	IC_50_ (15 μM) -glioma cells	-inhibition of TNFα-mediated NF-κB activation and sensitizing of cells to TNFα-induced apoptosis	Cozza et al., 2013 Dixit et al., 2012
4,5,6,7-tetrabromo-1H-benzimidazole **(TBI/TBBz/TBBi)**	IC_50_ (0.50 μM) -C6 (rat glioma cells) -T98G (glioblastoma cells) -SEGA (subependymal giant cell astrocytoma)	-modulation and transduction of many signalling pathways, including mTOR kinase-related pathways -inhibition of PIM, PKD1, HIPK2, DYRK1a kinase	Duncan et al., 2008 Kaminska et al., 2009 Pucko et al., 2019 Pagano et al., 2008
4,5,6,7-tetrabromo-1H-benzimidazole-2-N, N-dimethylamine **(DMAT)**	IC_50_ (0.14 μM) -T98G (glioblastoma cells) -LN229 glioblastoma cells -MCF-7 (human breast cancer cells) -KG-1 (human acute leukaemia myeloid cells) -H295R (human adrenocortical cancer cell line) -colorectal cancer	-activation of caspases 3, 7, and 8; increased expression of FasL and Fas; weakened membrane potential and mitochondrial function -induction of cell apoptosis	Duncan et al., 2008 Pucko et al., 2019 Kaminska et al., 2009 Koronkiewicz et al., 2013 Lawnicka et al., 2010 Tapia et al., 2006
4,5,6,7-tetrabromo-1H-benzotriazole **(TBB/TBBt)**	IC_50_ (0.50 μM) -T98G (glioblastoma cells) -CLL (chronic lymphocytic leukaemia cells)	-induction of cell apopotosis -inhibition of PIM family kinases including PIM1 and PIM3 -reduction in PTEN and phosphorylation kinase Akt	Duncan et al., 2008 Kaminska et al., 2009; Pucko et al., 2019 Andrzejewska et al., 2003 Pagano et al., 2008 Shehata et al., 2010
1-β-D-2′-deoxyribofuranosyl-4,5,6,7-tetrabromo-1H-benzimidazole **TDB/****K164**	IC_50_ (32 nM) -CEM (human T-lymphoblastoid cells) -HeLa (human cervical cancer cells)	-inhibition of PIM1, CLK2, DYRK1A kinases -induced apoptosis	Girardi et al., 2015 G. Cozza at al., 2014
5- (3-chlorophenylamino) benzo [c] [2,6] naphthyridine-8-carboxylic acid **CX-4945**	IC_50_ (0.3 nM) -human hematological malignancies -solid tumours -cholangiocarcinoma -medulloblastoma cell lines -acute lymphoblastic leukaemia glioblastoma	-may act synergistically with several anticancer drugs such as gemcitabine, cisplatin, and bortezomib against cholangiocarcinoma and acute lymphoblastic leukaemia -induction of apoptosis -synergistic action with gefitinib exerted a strong antiproliferative effect on glioblastoma	Zhou et al., 2017 D’Amore et al., 2020 Buontempo et al., 2016 Nitta et al., 2019 Zakharia et al., 2019 Rowse et al., 2017
N-(5-(3-Cyano-7-(cyclopropylamino)pyrazolo[1,5-a]pyrimidin-5-ylamino)-2-methylphenyl)propionamide **SGC-CK2-1**	IC_50_ (36 nM) U-87MG (glioblastoma cells)	showed no antiproliferative activity	Salvi et al., 2021
Isothiourea derivative (pentabromobenzylisothioureas) **ZKK**	IC_50_ (7–50 μM) -LN229 (glioblastoma cells) -C6 (rat glioma cells) -T98G (glioblastoma cells) -HL-60 (human promyelocytic leukaemia) -K-562l (human chronic erythromyeloblastic leukaemia)	-induction of apoptosis -combination of HBO with the ZKK3 increased cytotoxicity and made T98G cells more sensitive to antitumour effect -ZKK3 inhibited PIM, IGF-1R, IR, PKD1 kinases	Kaminska et al., 2009 Pucko et al., 2018 Koronkiewicz et al., 2013 Zembrzuska et al., 2019 Koronkiewicz et al., 2013
